# Supplementary material for: A Cancer-Specific Monoclonal Antibody against Podocalyxin Exerted Antitumor Activities in Pancreatic Cancer Xenografts
Source: Int J Mol Sci. 2023 Dec 21;25(1):161. doi: 10.3390/ijms25010161 (PMC10779310; doi:10.3390/ijms25010161)
Supplement: Supplementary file 1 [file ijms-25-00161-s001.zip › ijms-2767960-supplementary.pdf]

Supporting Information

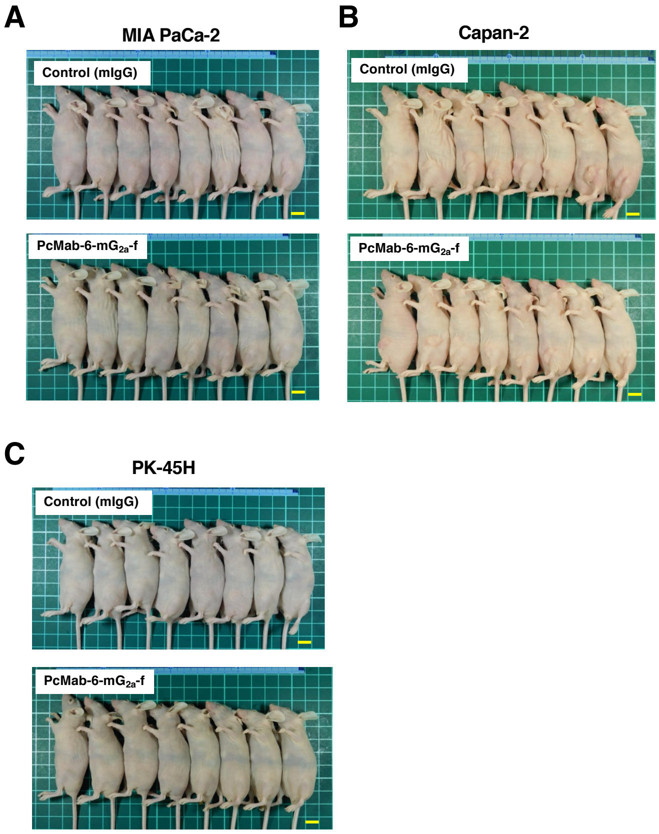

Supplementary Figure S1. Body appearance in MIA PaCa-2 (A), Capan-2 (B), and PK-45H (C) xenografts-implanted mice treated with control mIgG or PcMab-6-mG_{2a}-f on day 25 (PK-45H) or day 28 (MIA PaCa-2 and Capan-2) after cell implantation. Scale bar, 1 cm.

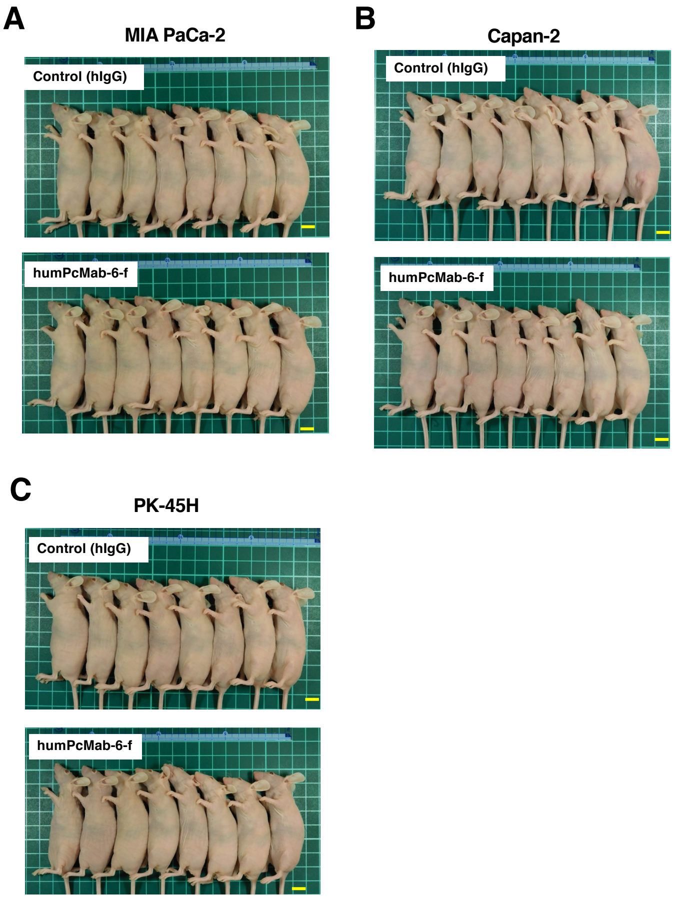

Supplementary Figure S2. Body appearance in MIA PaCa-2 (A), Capan-2 (B), and PK-45H (C) xenografts-implanted mice treated with control hIgG or humPcMab-6-f on day 21. Scale bar, 1 cm.

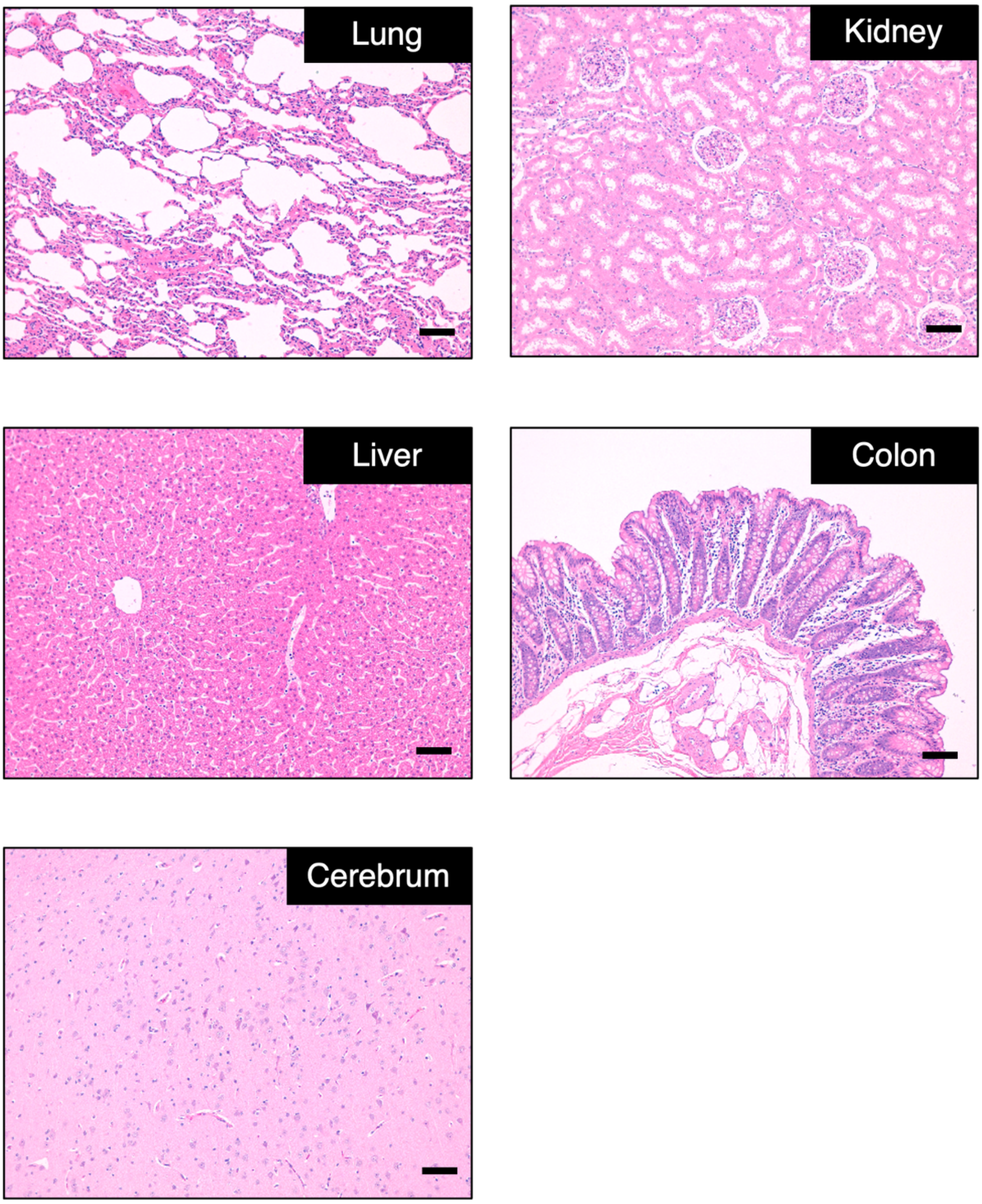

Supplementary Figure S3. The toxicity of chPcMab-6-f against cynomolgus monkey. A cynomolgus monkey was intravenously injected with chPcMab-6-f (20 mg/kg). After one week, the animal was sacrificed and the morphology of tissues (lung, kidney, liver, colon, and cerebrum) was investigated by hematoxylin and eosin staining. There was no abnormality in these tissues. Scale bar, 200 µm.
